# Differential Expression of PRAMEL1, a Cancer/Testis Antigen, during Spermatogenesis in the Mouse

**DOI:** 10.1371/journal.pone.0060611

**Published:** 2013-04-02

**Authors:** Bhavesh V. Mistry, Yaqi Zhao, Ti-Cheng Chang, Hiroshi Yasue, Mitsuru Chiba, Jon Oatley, Francisco Diaz, Wan-Sheng Liu

**Affiliations:** 1 Department of Animal Science, Center for Reproductive Biology and Health (CRBH), College of Agricultural Sciences, The Pennsylvania State University, University Park, Pennsylvania, United States of America; 2 National Institute of Agrobiological Sciences, Tsukuba, Ibaraki, Japan; 3 Division of Medical Life Sciences, Department of Biomedical Sciences, Hirosaki University Graduate School of Health Sciences, Hirosaki, Aomori, Japan; Baylor College of Medicine, United States of America

## Abstract

PRAME belongs to a group of cancer/testis antigens (CTAs) that are characterized by their restricted expression in normal gametogenic tissues and a variety of tumors. The *PRAME* family is one of the most amplified gene families in the mouse and other mammalian genomes. Members of the *PRAME* gene family encode leucine-rich repeat (LRR) proteins functioning as transcription regulators in cancer cells. However, the role of PRAME in normal gonads is unknown. The objective of this study is to characterize the temporal and spatial expression of the mouse *Pramel1* gene, and to determine the cellular localization of the PRAMEL1 protein during the mouse spermatogenesis. Our results indicated that the mouse *Pramel1* was expressed in testis only. The mRNA and protein expression level was low in the newborn testes, and gradually increased from 1- to 3-week-old testes, and then remained constant after three weeks of age. Immunofluorescent staining on testis sections with the mouse PRAMEL1 antibody revealed that PRAMEL1 was localized in the cytoplasm of spermatocytes and the acrosomal region of round, elongating and elongated spermatids. Further analyses on the testis squash preparation and spermatozoa at a subcellular level indicated that the protein localization patterns of PRAMEL1 were coordinated with morphological alterations during acrosome formation in spermatids, and were significantly different in connecting piece, middle piece and principal piece of the flagellum between testicular and epididymal spermatozoa. Collectively, our results suggest that PRAMEL1 may play a role in acrosome biogenesis and sperm motility.

## Introduction

Spermatogenesis is a complex biological process involving the mitotic proliferation of spermatogonia, the meiotic division of spermatocytes, and the morphogenic differentiation of spermatids to mature spermatozoa. During spermiogenesis, the round spermatids undergo dramatic morphological and cellular changes, including formation of the acrosome, elongation and condensation of the nucleus, formation of the flagellum, and disposal of unnecessary cytoplasm, to produce highly specialized and polarized spermatozoa [1], [2]. The acrosome is a Golgi-derived vesicle located at the anterior region of the sperm head [3]. It plays an essential role in fertilization by its involvement in the process of sperm-egg binding and penetrating the egg [4], [5]. The mammalian males carrying mutations that affect acrosome biogenesis are infertile or subfertile [4]–[7]. Acrosome biogenesis begins at the late pachytene spermatocyte phase of meiosis and continues throughout the first half of spermiogenesis [4], [5], [8]. Initially, proacrosomal vesicles are formed from Golgi apparatus and assembled in the perinuclear region, near one of the poles of the nucleus in pachytene spermatocytes. After completion of the meiotic division, these vesicles fuse with each other to form a single large acrosomal vesicle that attaches to the nuclear envelope of round spermatids and continues to enlarge by addition of Golgi-derived material [4], [8]. During maturation of the elongating spermatids, the Golgi ceases to contribute glycoproteins to the acrosome, and the acrosome-nucleus complex undergoes extensive morphological changes to acquire a shape characteristic of the given species' sperm [8].

The flagellum of a mammalian spermatozoon is an important and highly organized microtubule-based complex structure that provides motility required for the sperm to reach and fertilize an egg [9]. Structurally, it is divided into four major components: the connecting piece, the middle piece, the principal piece, and the end piece [10]. Assembly of the flagellum starts at the beginning of spermiogenesis. During flagellum biogenesis, a pair of centrioles moves to the opposite side of the developing acrosome in the perinuclear space of the round spermatid, where one of the two centrioles forms a flagellar axoneme. Subsequently, the axoneme protrudes from the spermatid, yielding the flagellum [8]. More than 400 proteins have been found in the flagellum, but only a fraction of the proteins have been characterized at the molecular level and shown to function as structural, metabolic or signaling proteins [11], [12].

Cancer/testis antigens (CTAs) are a group of proteins that are highly expressed in a wide variety of malignant tumors, but their expression in normal tissues is mostly restricted to testicular and ovarian germ cells [13]–[15]. Due to their characteristic expression pattern, CTAs are used as prognostic markers for a range of tumors and are also considered promising targets for cancer immunotherapy [16]–[18]. At least 70 CTA gene families involving more than 240 individual genes (or isoforms) have been identified to date [19]. Previous studies have suggested that CTAs, in general, have a role in both tumorigenesis and gametogenesis [14], [15], [20]. However, information regarding the function and precise cellular localization of CTAs in germ cells and cancer tissues is sparse compared to data on their expression and immunogenicity [13], [15].

Preferentially expressed antigen in melanoma (PRAME) is a member of the CTAs, and was originally identified as a tumor antigen expressed in melanoma cells, triggering an autologous cytotoxic T cell-mediated immune response [21]. *PRAME* is a multi-copy gene representing one of the most amplified gene families in eutherian mammals, with ∼ 90 copies in the mouse, ∼ 50 copies in the human and ∼ 30 copies in the bovine genome [19], [22], [23]. Interestingly, *PRAME* has been transposed to and amplified on the Y chromosome in the bovid lineage, suggesting a role in male reproduction [19]. Members of *PRAME* gene family encode LRR (leucine-rich repeat) proteins that fold into a horseshoe shape in their tertiary structure. The primary function of LRR proteins is to provide a versatile structural framework for the formation of protein–protein interactions in diverse molecular recognition processes, including signal transduction, cell adhesion, transcriptional regulation, RNA processing, apoptosis, immune response, and cell polarization [24]–[28]. Expression and function of PRAME has been extensively studied in a variety of cancer cells, and high PRAME expression correlates with cancer progression [28]–[30]. In melanoma cells, PRAME functions as a repressor of retinoic acid (RA) signaling through the interaction with RA receptors (RARs) and recruitment of polycomb proteins [27], [31]. Recent studies have also suggested that PRAME functions as a transcription activator. Costessi *et al.* (2011, 2012) reported that PRAME is a component of Cullin2 based E3 ubiquitin ligase and is required to recruit Cullin2 ubiquitin ligase to the EKC/KEOPS complex. This complex is associated with the active promoters regulated by the *nuclear transcription factor Y* (*NFY*) [29], [32]. We hypothesize that PRAME also plays a role in spermatogenesis. As an initial step to test our hypothesis and to dissect the molecular role of the PRAME gene family in reproduction, we chose to work on the mouse *Prame-like 1* (*Pramel1*) gene, a representative of the *Prame* gene family, which is homologous to the human *PRAME,* and is located on the mouse chromosome (MMU) 4. In this work, we characterized the *Pramel1* gene with respect to its mRNA and protein expression pattern and cellular localization during testis development and spermatogenesis.

## Results

### Temporal and spatial expression analysis of the mouse *Pramel1* in testis

**Figure 1:**
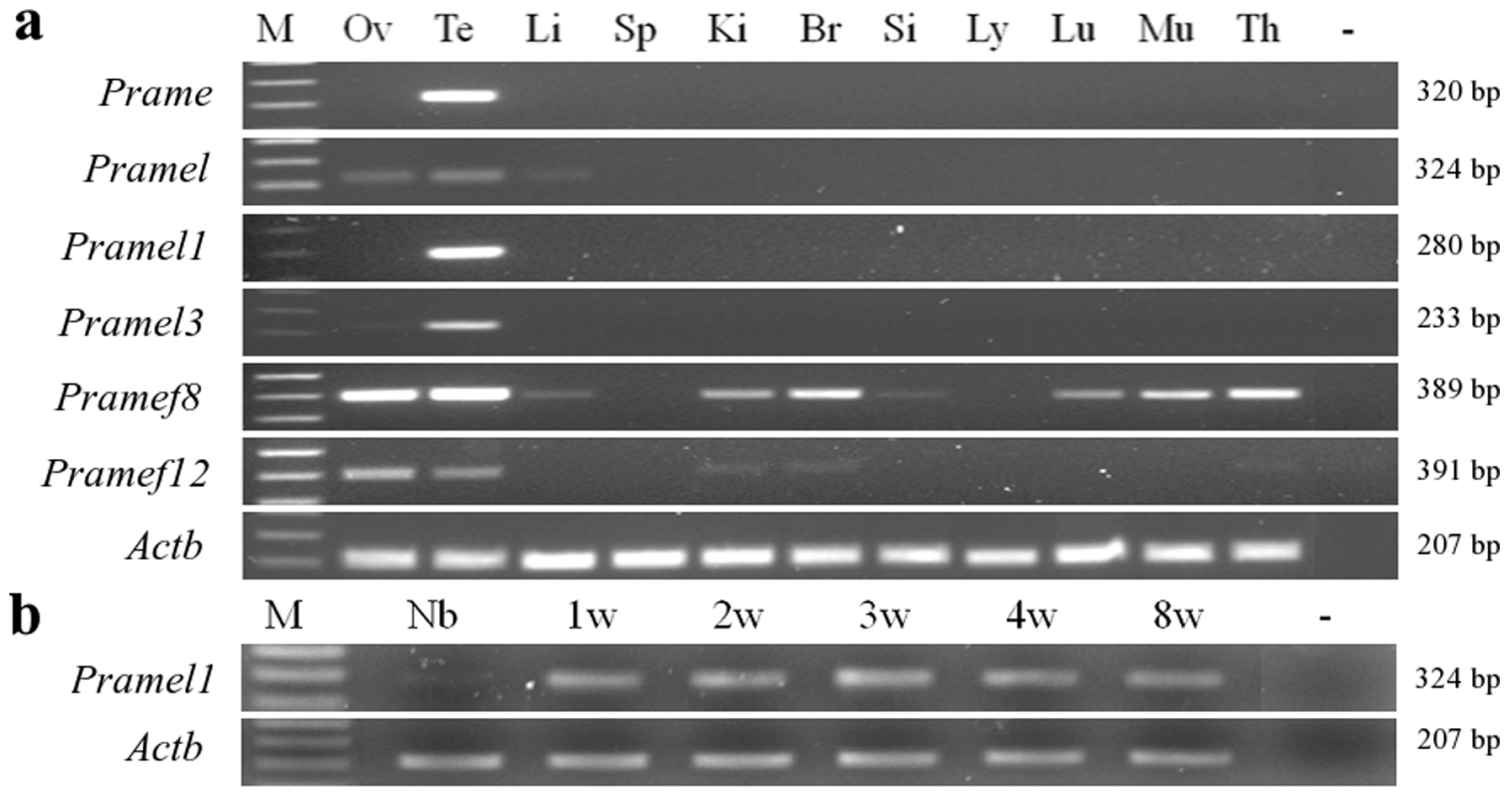
Expression analysis of the mouse *Prame* gene family by RT-PCR. **a.** Spatial expression analysis of six *Prame* paralogs among 11 different tissues of adult mice. *Prame* and *Pramel1* were expressed specifically in testis, whereas *Pramel, Pramel3, Pramef8* and *Pramef12* were expressed predominantly in gametogenic tissues with a low level of expression in liver, kidney, brain, small intestine, lung, muscles and thymus. **b.** Temporal expression analysis of *Pramel1* during testis development. Low expression was observed in the newborn testis, while constant high expression was observed in testes from 1- to 8-week-old mice. *Actb* was used as a positive control. M: marker; Ov: ovary; Te: testis; Li: liver; Sp: spleen; Ki: kidney; Br: brain; Si: small intestine; Ly: lymph node; Lu: lung; Mu: muscles; Th: thymus; -: negative control.

The expression pattern of the mouse *Pramel1* gene (acc. no. NM_031377) was examined in 11 tissues of adult mice by RT-PCR. For comparative purposes, five other paralogs from the mouse *Prame* family (X-linked *Prame*, *Pramel3*, and *Pramel*, and MMU4-linked *Pramef8* and *Pramef12*) were also analyzed (Table 1). The results indicated that all six paralogs were highly expressed in testis, with varying expression levels in the other tissues (Fig. 1). The *Pramel1* and *Prame* mRNAs were specifically expressed in testis, whereas *Pramel*, *Pramel3*, *Pramef8*, and *Pramef12* mRNAs were predominantly expressed in testis with a low expression in ovary, liver, spleen, kidney, brain, lung, muscle and thymus (Fig. 1a). To further examine temporal and spatial expressions of *Pramel1,* we performed a time course study during testis development. Our RT-PCR results indicate that, while a lower level the *Pramel1* mRNA was expressed in the newborn testis, a higher level of constant expression was detected in 1-week- through 8-week-old testes (Fig. 1b).

**Table 1:** Oligo primers for RT-PCR analysis.

Gene	Accession number	Primer sequence (5′ to 3′)	Product length (bp)
*Prame*	NM_029459	AAGGACGGTGTCTGAGTGTG	320
		TGAAGACTCTCAACCATGGC	
*Pramel*	XM_885719	TGGCAGTTGATAAGGCTTCC	324
		AGATTCAGCCGCTTGAGGTG	
*Pramel1*	NM_031377	TCTGCTCTGGATGACATACC	280
		GGCAACCTGTTCCACAGCTT	
*Pramel3*	NM_031390	TGTTCACTGCTGCCTTCACG	233
		GACATACGGTCTTGCAGCCA	
*Pramef8*	NM_172877	TCTTCTCTGCAGTACCTTCC	389
		GAGGCACAGGTCAGTGACCA	
*Pramef12*	NM_02994	TGTGAAGATGAGCTTGCGTG	391
		CTGGAGCGCAAGATGTTGTC	
*Actb*	NM_007393	CTAGACTTCGAGCAGGAGAT	207
		GGATGTCAACGTCACACTTC	

**Figure 2:**
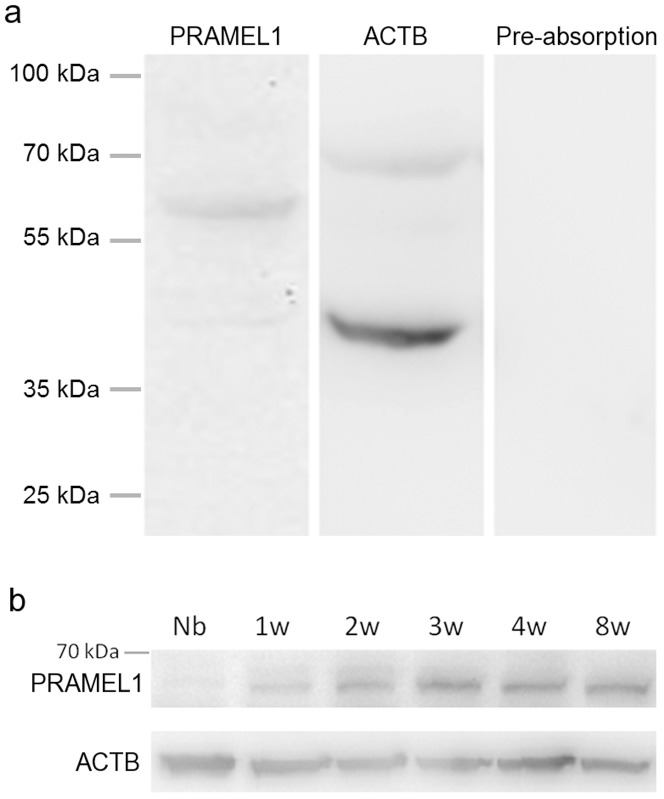
Expression analysis of the mouse PRAMEL1 protein in testes. **a.** Western blot analysis of PRAMEL1. Protein extracts from adult mouse testis were subjected to western blot analysis using a mouse PRAMEL1 antibody. An immune-reactive protein with an expected molecular weight of ∼ 57 kDa was detected in the testis, and a very minor band of ∼ 42 kDa was also observed. As a positive and loading control a monoclonal ACTB antibody was used, and an expected immune-reactive protein of 42 kDa was observed. As a negative control, we performed a pre-absorption control by the peptide used to generate the PRAMEL1 antibody, and no band was detected. **b.** Time course analysis of the PRAMEL1 protein expression during testis development. Protein extracts from newborn (Nb), 1-week (1w), 2-weeks (2w), 3-weeks (3w), 4-weeks (4w) and 8-weeks (8w)-old testes were subjected to western blotting with the mouse PRAMEL1 antibody. The expression level of the PRAMEL1 was gradually increased from Nb to 3w and then remained constant after 3-weeks of age. Numbers on the left indicate the molecular weights of standard proteins.

To determine whether the PRAMEL1 protein is expressed in testis, a western blot analysis was performed on whole testis lysate from mature mice using a PRAMEL1-specific antibody. As shown in Figure 2a, the PRAMEL1 antibody identified a specific band with a molecular weight of ∼ 57 kDa, which is the predicted molecular weight for the mouse PRAMEL1 protein (acc. no. NP_113554). In addition, a very minor band (∼ 42 kDa) was observed (Fig. 2a). To test the specificity of the antibody, we performed a pre-absorption control with the peptide used to generate the PRAMEL1 antibody, in which no band was detected (Fig. 2a), suggesting that the antibody is PRAMEL1-specific and the minor band requires further investigation. A time course analysis of the PRAMEL1 protein further indicated that its expression in testes was age-dependent (Fig. 2b). The protein expression level was very low at the newborn stage (Fig. 2b), consistent with the low level of transcription detected by RT-PCR (Fig. 1b). The expression was increased gradually from 1- to 3-week-old testes, and then remained constant after three weeks of age (Fig. 2b). As a positive control, a monoclonal β-actin (ACTB) antibody was used to detect ACTB, and an expected protein of 42 kDa was identified (Fig. 2a, 2b).

### Localization of the PRAMEL1 protein during spermatogenesis

**Figure 3:**
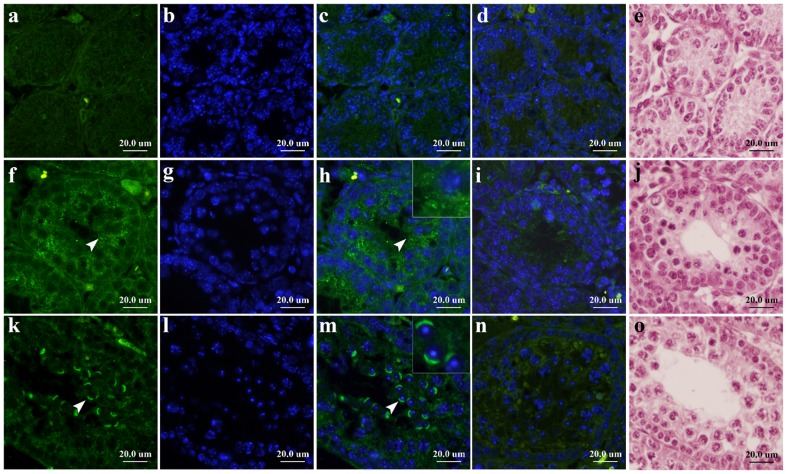
Immunofluorescent localization of the mouse PRAMEL1 during early testis development. Cross-sections from 1-week (**a**–**e**), 2-week (**f**–**j**) and 3-week (**k**–**o**)-old mouse testes were stained with the mouse PRAMEL1 antibody. Although there was no specific signal observed across the seminiferous tubules in 1-week-old testis, specific staining (green) was observed in cytoplasm of spermatocytes (**f**, **h**) in 2-week-old testis and the perinuclear region of round spermatids (**k**, **m**) in 3-week-old testis. Panels **a**, **f**, **k** are the PRAMEL1 antibody staining. Panels **b**, **g** and **l** are counterstained with DAPI to visualize nuclei. Panels **c**, **h** and **m** are merged images for the PRAMEL1 and DAPI staining. The arrowheads point to an area with described signals. Panels **d**, **i** and **n** are peptide-preabsorbed antibody staining as negative control. Panels **e**, **j** and **o** are H&E staining to show the structure and cell types of the seminiferous tubules. Magnification is ×400. Scale bar  =  20 µm.

We investigated the cellular, spatial and temporal expression of the PRAMEL1 protein during germ cell development by indirect immunofluorescence microscopy. Initially, we examined the PRAMEL1 protein localization on cross-sections of testes in young mice, from newborn to 3-weeks of age. As shown in Fig. 3, there was no specific staining pattern observed for the PRAMEL1 antibody across the section in 1-week-old testis (Fig. 3a, c), though weakly unspecific background staining was observed for both the PRAMEL1 antibody (Fig. 3a, c) and pre-absorbed peptide control (Fig. 3d). This might be a consequence of relatively low expression of the transcript and protein at this stage (Fig. 1b and 2b). Compared to the pre-absorbed peptide control where some non-specific staining was observed (Fig. 3i), specific staining was seen for the PRAMEL1 antibody mainly in the cytoplasm of spermatocytes in 2-week-old testis (Fig. 3f, h). When the mice reached 3 weeks of age, a very unique and strong staining was detected by the PRAMEL1 antibody in the perinuclear region of round spermatids (Fig. 3k, m), where a cap-like structure on the nuclear surface was observed (Fig. 3m).

**Figure 4:**
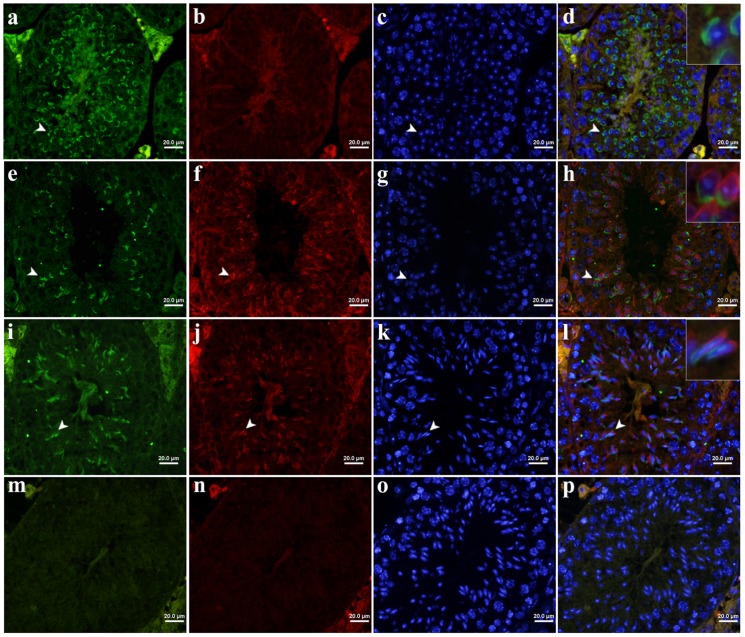
Immunofluorescent localization of the mouse PRAMEL1 in mature testis. Cross-sections from adult (≥ 2 months old) mouse testes were double-stained with the mouse PRAMEL1 (green) and TUBA (red) antibodies. The PRAMEL1 staining was observed in round (**a**), elongating (**e**) and elongated (**i**) spermatids during spermiogenesis. The TUBA staining was observed in elongating (**f**) and elongated (**j**) spermatids, but not in round spermatids (**b**). All sections were counterstained with DAPI (Panels **c**, **g**, **k** and **o**) to visualize nuclei. Panels **d**, **h**, **l** and **p** are merged images for PRAMEL1, TUBA and DAPI staining. The arrowheads point to an area with described signals. The insets in **d**, **h**, and **l** are enlarged image for arrowhead-marked areas (also see Fig. 5a, c, e and g). Panels **m** and **n** are secondary antibody controls for FITC-labeled donkey anti-goat IgG and TRITC-labeled donkey anti-mouse IgG, respectively. Magnification is ×400. Scale bar  =  20 µm.

**Figure 5:**
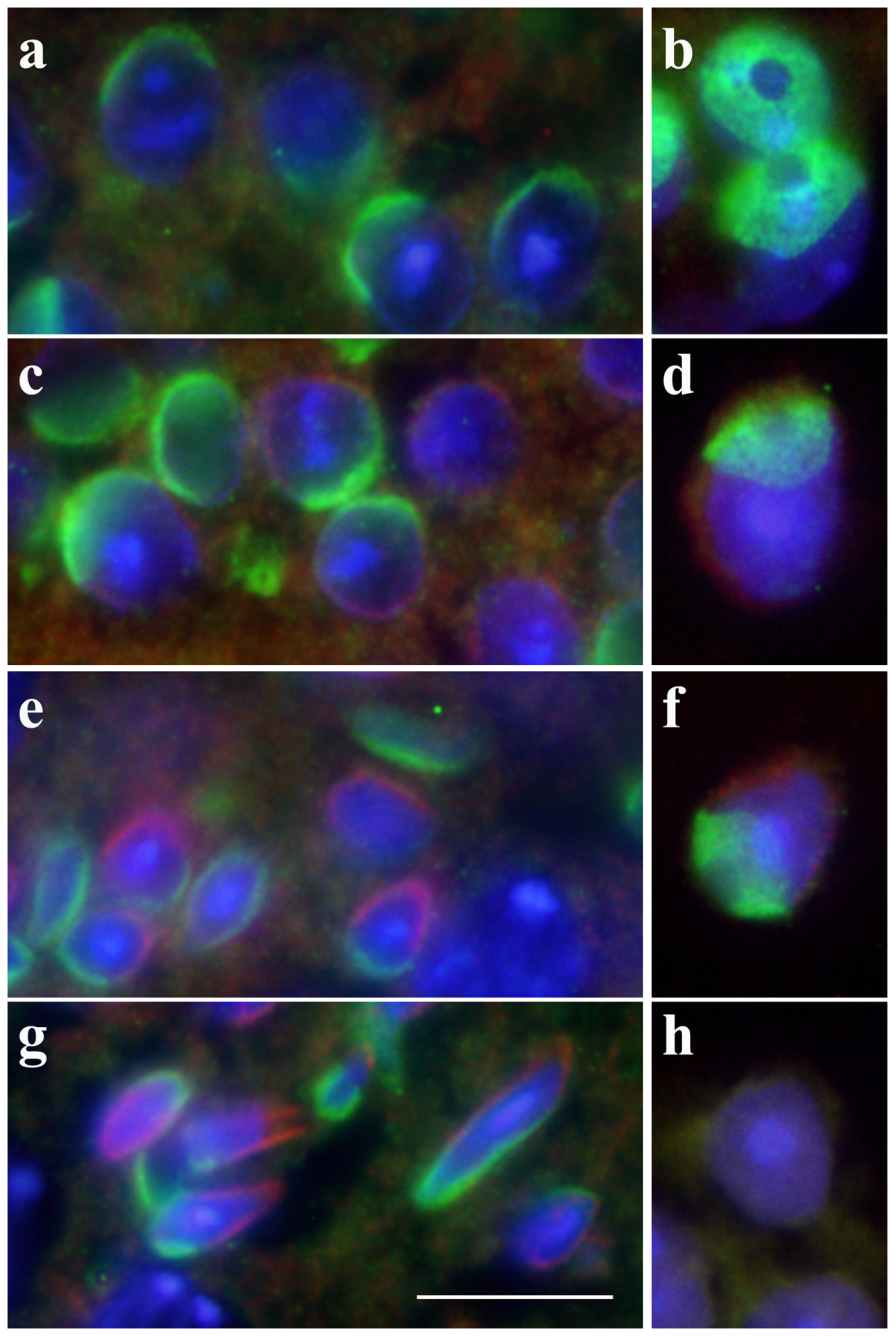
Immunofluorescent localization of the mouse PRAMEL1 in spermatids. Testis sections (**a**, **c**, **e** and **g**) and squash preparations of seminiferous tubules (**b**, **d,** **f** and **h**) were stained with anti-PRAMEL1 (green), anti-TUBB (red) and DAPI (blue). Merged images for the triple-staining in round spermatids (**a** and **b**), elongating spermatids (**c**, **d**, **e** and **f**) and elongated spermatids (**g**) are shown. The PRAMEL1 staining was observed in the acrosomal vesicle region of all the developing spermatids and the TUBB staining was observed in the manchette of elongating and elongated spermatids. As a secondary antibody control testis squash preparation (**h**) was stained with FITC-labeled donkey anti-goat IgG and TRITC-labeled donkey anti-mouse IgG. Magnification is ×1000. Scale bar  =  10 µm.

To further examine whether the cap-like structure on the nuclear surface of round spermatids is at the anterior or posterior side, the mouse PRAMEL1 antibody together with the β-tubulin (TUBB) antibody that is specifically stained in the posterior side of spermatids [33], were applied to cross-sections of testes from adult mice. Under low magnification (×400), as the differentiation of the seminiferous epithelium proceeds, PRAMEL1 can be seen in the perinuclear region of post-meiotic germ cells (round, elongating and elongated spermatids) (Fig. 4a, e, i), whereas TUBB was seen at the opposite side of the PRAMEL1 staining in the elongating and elongated spermatids (Fig. 4e, i), indicating that the PRAMEL1 protein was located at the anterior side (*i.e.* acrosome) of the nuclear region in mouse spermatids. A closer examination of the immunofluorescently-stained testis sections at higher magnification (×1000) showed that the PRAMEL1 protein was first seen in round spermatids, where it was closely associated with a cap-like structure on the nuclear surface (Fig. 5a, b). Compared to the testis section, the squashed preparation of round spermatids offered more details about the staining pattern, where PRAMEL1 was mainly observed in the acrosomal vesicle, with little or no staining in the acrosomal granule region (Fig. 5b). This pattern coincided with the formation of the acrosomal vesicle that spreads in a thin layer over the pole of the nucleus to form the acrosomal cap. At this stage, no staining was observed for TUBB because the flagellum had not yet formed (Fig. 4a–d; Fig. 5a, b). Notably, the pattern of PRAMEL1 localization changed according to morphological alterations occurring in the acrosome and nucleus during spermiogenesis (Fig. 4a, e, i; Fig. 5a–g). When the round spermatids begin to elongate and the flagellum starts to develop, TUBB was seen at the opposite side of the PRAMEL1 staining in the perinuclear region, marking the position of manchette development and flagellum formation (Fig. 4e, i; Fig. 5c–g). With the progress of spermatid morphogenesis, the PRAMEL1 protein staining expanded over approximately 1/3 of the nuclear surface, mimicking acrosome development (Fig. 5a–g). Therefore, our results clearly indicate that the PRAMEL1 protein is associated with the acrosome in spermatids. As negative controls, sections were stained either with the primary antibody pre-absorbed with the respective peptide or with the secondary antibody only. No fluorescence signal was detected in the control sections, indicating that non-specific staining was minimal (Fig. 4m–p; Fig. 5h).

### Localization of PRAMEL1 in testicular and epididymal spermatozoa

**Figure 6:**
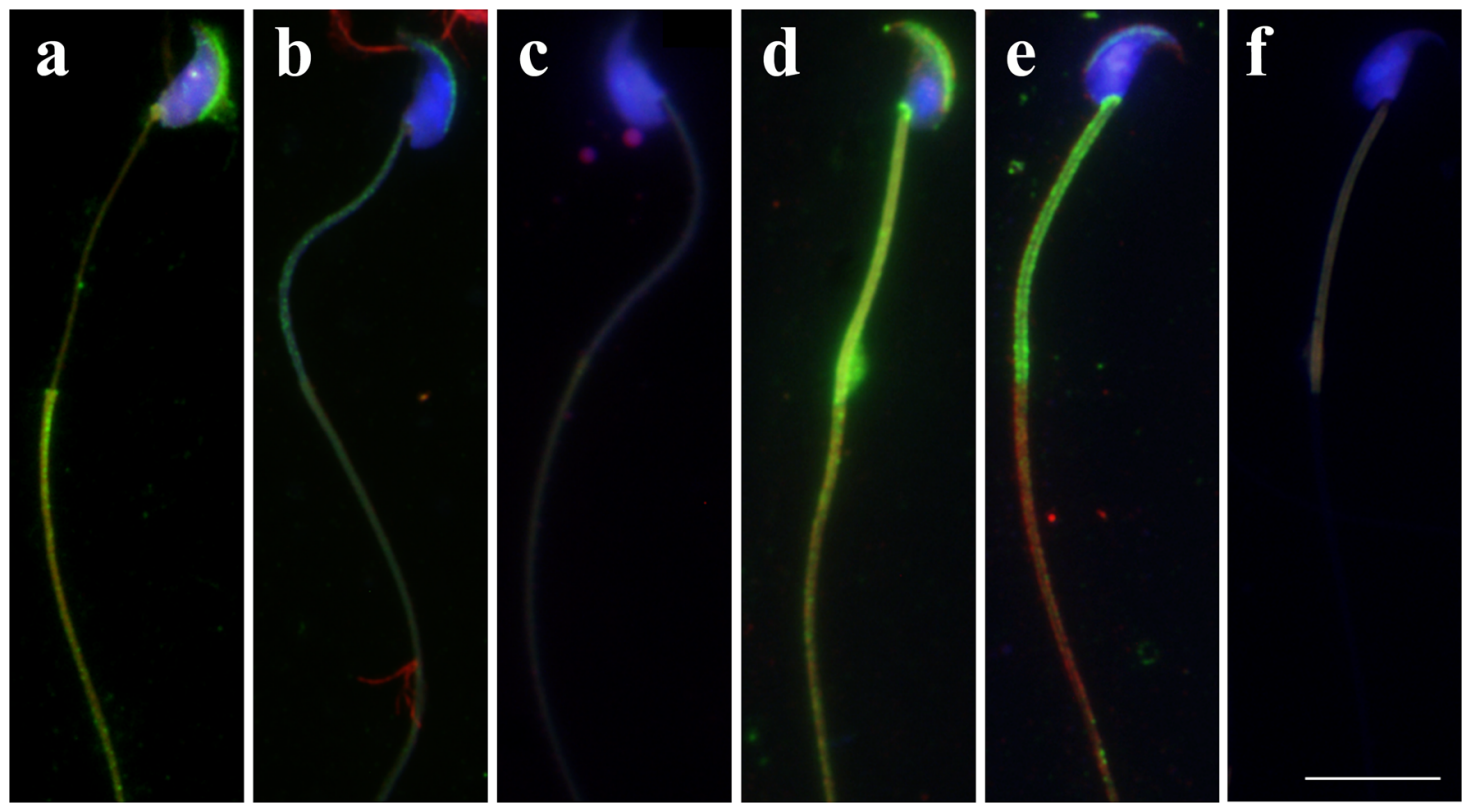
Immunofluorescent localization of the mouse PRAMEL1 in spermatozoa. Mouse spermatozoa collected from squash preparations of seminiferous tubules (**a**, **b** and **c**) and epididymis (**d**, **e** and **f**) were stained with the PRAMEL1 (green), TUBA (red) antibodies and DAPI (blue). The PRAMEL1 staining was observed in the acrosome region of testicular and epididymal spermatozoa (**a**, **b, d** and **e**). In testicular spermatozoa (**a**) PRAMEL1 was also detected in the principal piece of the sperm flagellum, whereas in epididymal spermatozoa (**d** and **e**) the PRAMEL1 staining was observed predominantly in connecting piece, middle piece and cytoplasmic droplet. As a secondary antibody control the testicular (**c**) and epididymal (**f**) spermatozoa were stained with FITC-labeled donkey anti-goat IgG and TRITC-labeled donkey anti-mouse IgG. Magnification ×1000. Scale bar  =  10 µm.

We further investigated the subcellular distribution of PRAMEL1 in testicular/epididymal spermatozoa by performing indirect immunofluorescence staining, using the PRAMEL1- and α-tubulin (TUBA)-specific antibodies. The spermatozoa were collected from squashed seminiferous tubules of mature testes, and caput, corpus and cauda regions of the epididymis. The results revealed that, similar to staining in spermatids, PRAMEL1 is mainly localized to the anterior acrosome region of spermatozoa (Fig. 6a, b, d, e; Table 2). As different staining patterns were observed between testicular and epididymal spermatozoa, over 200 spermatozoa from each group (Table 2) were examined to study the detailed localization of the PRAMEL1 protein in sperm cells. We found that all testicular spermatozoa (100%) showed acrosomal localization of PRAMEL1, while only 86.5% of epididymal spermatozoa were positive for PRAMEL1 in the acrosomal region (Table 2). Furthermore, PRAMEL1 was observed on the principal piece of the flagellum (41%) in testicular spermatozoa (Fig. 6a, Table 2), whereas it was observed in the connecting piece (96%) and middle piece (87.9%) in epididymal spermatozoa (caput, corpus, and cauda) (Fig. 6a, b, d, e; Table 2). PRAMEL1 staining was also seen in cytoplasmic droplets of epididymal spermatozoa (Fig. 6d). We found that 55% of epididymal spermatozoa had cytoplasmic droplets located at the distal end of the middle piece. Around 73% of those cytoplasmic droplet-bearing spermatozoa showed the PRAMEL1 signal in the cytoplasmic droplet (Fig. 6d; Table 2). We also observed that the percentage of cytoplasmic droplet-bearing spermatozoa varied among the three mice studied. For both testicular and epididymal spermatozoa, no PRAMEL1 staining was observed in the annulus region of the flagellum. TUBA was localized in the anterior acrosomal region of sperm heads, and the middle piece and principal piece of sperm flagellum of epididymal spermatozoa (Fig. 6a, b, d, and e). No signal was observed when primary antibodies were omitted during the staining, suggesting minimal non-specific binding of fluorescently-labeled secondary antibodies (Fig. 6c, f).

**Table 2:** PRAMEL1 staining in different organelles of spermatozoa.

Sperm origin	Number of sperm	Acrosome	Connecting piece	Middle piece	Principal piece	Cytoplasmic droplet
Testicular spermatozoa	202	100%	0	0	41.1%	0
Epididymal sperm	223	86.5%	96.0%	87.9%	0	40.2%*

* This was calculated based on 55% of the analyzed epididymal spermatozoa had cytoplasmic droplets and 73% of those cytoplasmic droplet-bearing spermatozoa showed the PRAMEL1 signal in their cytoplasmic droplets.

## Discussion

Although PRAME has been extensively studied in tumorigenesis and cancer biology, and is being used as a biomarker for cancer diagnosis and antigen-specific cancer immunotherapy [28]–[32], [34], the function of the PRAME gene family in gametogenesis and reproduction has not been characterized. In the present study, we characterized the mRNA and protein expression of the mouse *Pramel1* gene, a member of the *Prame* gene family, during testis development and spermatogenesis. Our results clearly indicated that PRAMEL1 is expressed in the cytoplasm of the spermatocytes and the acrosome and flagellum of spermatids and spermatozoa, which provide the first insight into the potential function of the *Prame* gene family in spermatogenesis. In order to understand the biological role(s) of this entire gene family and the relationship between the *Pramel1* and other family members, we retrieved expressional data for a total of 10 *Prame* paralogs from the Gene Expression Omnibus (GEO) (http://www.ncbi.nlm.nih.gov/sites/geo) (Table 3) [35]–[40]. Combined with the RT-PCR data obtained from this work, we were able to cluster these paralogs into three major groups based on their spatial and temporal expression profiles: group I, expressed only in male gonad and germ cell (*Prame* and *Pramel1*); group II, expressed only in female gonad and germ cell (*Oog1-3*); and group III, predominantly expressed in both male and female gonads (*Pramel, Praeml3, Pramef8, Pramef12,* and *Oog4*) (Table 3). Collectively, the divergent expression patterns of the *Prame* paralogs during gametogenesis in both male and female indicate that different members of the *Prame* gene family may be involved in different developmental stages of gametogenesis and embryogenesis.

**Table 3:** Expression profiles of the *Prame* gene family*.

Gene	Chr. No.	Embryo				Male						Female		
		Embryonic stem cell	One-cell	Four-cell	Gonad (d11)	Testis	SSC	Type A and B Spermatogonia	Pachytene spermatocyte	Round spermatid	Sperm	Ovary	Placenta	Oocytes
Prame	X				+	+				+		−		
Pramel	X					+						+		
Pramel3	X					+	+					+		
Pramel1	4					+					+	−		
Pramef8	4	+			+	+		+	+			+	+	
Pramef122	4	+			+	+		+				+	+	
Oog1	4		+	+	−							+		+
Oog2	4		+	+								+		+
Oog3	4		+	+								+		+
Oog4	4		+	+		+						+		+

* +: expression detected; −: expression undetected; blank: expression data unavailable

It has been reported in previous studies that different CTAs are involved in different stages of spermatogenesis. Some of which are expressed exclusively in one stage, such as SYCP1 (*synaptonemal complex protein 1*, also known as HOM-TES-14), which is restricted to meiotic prophase of spermatocytes, and SP17 (*sperm protein 17*), which is expressed in the mature spermatozoa only [41], [42]. Other CTAs such as CTAG1 (*cancer/testis antigen 1*, also known as NY-ESO-1) and TRO (*trophinin*, also known as *magphinin* or MAGE-D3), are expressed in several stages of spermatogenesis. CTAG1 is expressed in spermatogonia through pachytene spermatocytes [43], [44], while TRO is expressed in almost all types of germ cells from spermatogonia to mature spermatozoa [45]. In the present study, we demonstrated that, like the *Tro* gene, the mouse *Pramel1* is expressed broadly in different types of germ cells during spermatogenesis. The PRAMEL1 protein was first detected, though at a relatively low level, in the newborn testes by Western blotting, suggesting the expression of PRAMEL1 in spermatogonia. A gradually increased level of PRAMEL1 was detected in testes at 1-, 2-, 3-weeks of age and maintained at the higher level in mature testes. This increase in protein expression coincides with the increase in RNA expression from newborn to 1-week-old testes, and could be explained by an up-regulation of the gene expression, or alternatively, by an increase in the number of germ cells as there is dramatic germ cell proliferation before the first wave of spermatogenesis.

Unlike the cancer cells in which the PRAME protein was localized in the nucleus, the mouse PRAMEL1 was first seen in the cytoplasm of spermatocytes in 2-week-old testes in this study. A dominant expression of the PRAMEL1 protein was then observed in the anterior region of (round, elongating and elongated) spermatids, coordinating with the morphological alterations of the acrosome, which suggests that the *Pramel1* plays a role in acrosome development. Furthermore, we observed that PRAMEL1 was differentially distributed along different parts of the sperm flagellum between testicular and epididymal spermatozoa. As the spermatozoa gain motility during the transition from testis to the caudal region of epididymis, the differential localization of PRAMEL1 along different parts of the flagellum during the sperm transition may be an indication that PRAMEL1 is involved in the sperm maturation and motility (see discussion below).

Earlier studies in cancer cells demonstrated that PRAME functions either as a transcription suppressor of RA signaling [27], or as a transcription activator on promoter regions bound by NFY [29], [32]. However, our data suggest that *Pramel1* may not participate in transcription regulation through RA- and NFY-mediated mechanisms because (1) PRAMEL1 is localized to the cytoplasm of germ cells during spermatogenesis, and (2) most importantly, transcription is shut down during mid-spermiogenesis [27], [29], [31] and spermatozoa are terminally differentiated cells essentially devoid of transcriptional and translational activity [46].

It is worth noting that two LRR-containing, testis-expressed proteins have been implicated in spermiogenesis, namely LRRC67 (*leucine-rich repeat containing 67*, also known as testis LRR or TLRR) and PPP1R7 (*protein phosphatase 1, regulatory (inhibitor) subunit 7*, also known as Sds22) [47], [48]. Both proteins interact with a central phosphatase, *protein phosphatase 1* (PP1), which serves as a hub gene in several regulatory processes [46]–[48]. The LRRC67 protein interacts with PP1 to form a complex involved in cytoskeleton rearrangement in male germ cells [49]. The LRRC67 protein also interacts with a kinesin-related molecular motor, KIFC1 (*kinesin family member C1*), to transport molecules along the microtubules in the manchette [33], [50]. Similarly, the interaction of PP1 and PPP1R7 has also been implicated in sperm development. The dimerization of PPP1R7 and PP1γ2 leads to a decline of phosphatase activity of PP1γ2 and subsequently induces motility of caudal spermatozoa [51], [52]. Due to similar structural and expression patterns, we predict that PRAMEL1 may interact with PP1γ2 to transport molecules along the microtubules in the sperm flagellum. It remains to be established whether PRAMEL1 performs the similar function in acrosome formation and sperm motility.

## Materials and Methods

### Animals

C57BL/6J mice were bred in our colony at the Pennsylvania State University mouse facility, and a total of six pairs of breeders were set up. Testes were removed from mice at 0 day (newborn), 1 week, 2 weeks, 3 weeks, 4 weeks and 8 weeks of age. At least three animals (biological replicates) were used for each age. Testes, epididymis and other tissues from the adult male breeders and ovaries from the female breeders were collected after the breeding period. All animal procedures were approved by the Pennsylvania State University Institutional Animal Care and Use Committee (IACUC).

### Preparation of testicular tissues

For testis histology, whole testes were dissected out of adult mice and fixed by incubation overnight in Bouin solution (Sigma-Adrich, St. Louis, MO) at 4 °C. Tissues were then washed in 70% ethanol, dehydrated, and embedded in paraffin blocks. The paraffin-embedded tissues were sectioned at 4 µm using a microtome and mounted on glass slides.

Squashed testis samples were prepared as described previously [53]. Briefly, the adult testes were excised and transferred to a glass Petri dish containing ice-cold PBS (140 mM NaCl, 2.6 mM KCl, 6.4 mM Na_2_HPO_4_, and 1.4 mM KH_2_PO_4_, pH 7.4). After removing the tunica albuginea, seminiferous tubules were transferred to a new Petri dish containing PBS. Using fine forceps, the tubules were gently pulled apart and a small piece of single tubule was cut. The piece of seminiferous tubule was transferred to a new Petri dish and 15–30 µl of 0.1 M sucrose solution prepared in PBS was added. The cells were released from the tubule by tweezing apart the tubule and re-suspending it in the sucrose solution by pipetting up and down. Subsequently, the cells were transferred to slides predipped in 1% paraformaldehyde and 0.15% Triton-X100. Slides were dried and processed for immunofluorescence staining or frozen at −80 °C for later use.

### Isolation of epididymal sperm

Spermatozoa were collected by mincing caput, corpus and cauda epididymal sections from adult mice into 37 °C-equilibrated PBS and incubated for 5 min. Sperm were washed by centrifugation (300 × g, 5 min) and re-suspended in cold 0.1 M sucrose solution in PBS. Sperm smears were prepared on slides pre-dipped in 1% paraformaldehyde and 0.15% Triton-X100. Slides were dried at room temperature and processed for immunofluorescence staining as described below.

### RNA extraction and RT-PCR

Total RNA was extracted from 11 different tissues (ovary, testis, liver, spleen, kidney, brain, small intestine, lymph node, lung, muscles and thymus) of adult mice using TRIzol reagent (Invitrogen, Carlsbad, CA) according to the manufacturer's instructions. Total RNA of testes from young pups were extracted using RNeasy Mini Kit (Qiagen, Valencia, CA). The RNA samples were treated with DNase I (Ambion, Austin, TX) and reverse transcribed using the Superscript^TM^ III First-Strand Synthesis System (Invitrogen, Carlsbad, CA) as described by the manufacturer. RT-PCR was performed in 20 µl of a solution of 10 ng cDNA, 200 µM dNTPs, 1.5 mM MgCl_2_, 2.5 µM of each primer, 1 unit hot-star Taq DNA polymerase (Qiagen, Valencia, CA). The PCR conditions were: 95 °C for 15 min followed by 35 cycles each of 95 °C for 50 s, 55–65 °C for 50 s and 72 °C for 50 s, with a final extension at 72 °C for 10 min. Products were resolved on 1.5% agarose gels with ethidium bromide in 1×TAE buffer. Sequence-specific primers used for PCR amplification of mouse *Prame* orthologs, including three X-linked genes [*Prame* (NM_029459), *Pramel* (XM_885719) and *Pramel3* (NM_031390)] and three MMU4-linked genes [*Pramel1* (NM_031377), *Pramef8* (NM_172877) and *Pramef12* (NM_029948)], are shown in Table 1.

### PRAMEL1 antibody

A PRAMEL1 antibody (termed as PRAME like-1 (S-16), Catalog # sc-34513) was purchased from Santa Cruz Biotechnology (Santa Cruz, CA). PRAME like-1 (S-16) is an affinity-purified goat polyclonal antibody raised against a peptide of PRAME like-1 of mouse protein (accession no. NP_113554). The peptide for antibody production was chosen from the C-terminus region between 350-400 amino acid (aa) with a protein sequence similarity ≤ 58% to all other members of the mouse *Prame* gene family (NCBI Blastp analysis).

### Western blotting

Protein was isolated from mouse testes using RIPA-buffer containing proteinase and phosphatase inhibitors (sc-24948, Santa Cruz Biotechnology, Santa Cruz, CA). Total protein concentration was measured using BCA protein assay kit (product # 23227, Thermo Scientific Pierce, Rockford, IL), according to manufacturer's instruction. Total protein was re-suspended in SDS-sample buffer and boiled for 5 min at 95 °C. Equal amount (40 µg) of protein was electrophoresed on 10% polyacrylamide gels and transferred to PVDF membranes using Bio-Rad mini trans-blot modules according to manufacturer's instruction. Membranes were blocked for 1 h at room temperature in TBST buffer (10 mM Tris/HCl, pH 7.4, 150 mM NaCl, 0.05% Tween 20) containing 5% non-fat milk and incubated overnight at 4 °C with the PRAMEL1 antibody diluted (1:200) in blocking solution containing 5% non-fat milk. For control, membranes were incubated with a monoclonal ACTB antibody (Cat. no. A5441, Sigma-Aldrich, St. Louis, MO). Membranes were washed 3 times for 10 min with TBST and incubated for 1 h with a donkey anti-goat IgG-HRP (sc-2020, Santa Cruz Biotechnology, Santa Cruz, CA) or goat anti-mouse IgG-HRP (sc-2005, Santa Cruz Biotechnology, Santa Cruz, CA) at a dilution of 1:5000 prepared in blocking solution. Bound secondary antibodies were detected using the SuperSignal West Femto Chemiluminescent Substrate (product # 34096, Thermo Scientific Pierce, Rockford, IL). The peptide (sc-34513 P) used for making the PRAMEL1 antibody was applied to confirm the antibody specificity. The antibody and a five-fold (by weight) excess of peptide were incubated together for 2 hours at room temperature, which was analyzed by western blotting.

### Indirect immunofluorescence of testes and spermatozoa

Testis cross-sections (4 µm thick) were deparaffinized in xylene and hydrated in a graded series of alcohols. After washing in PBS, the sections were boiled for 20 min in 0.01 M sodium citrate buffer (pH 6.0) containing 0.05% Tween-20 using a microwave oven. Sections were then washed three times in PBS for 5 min each and blocked for non-specific antibody binding by incubating with 10% normal donkey serum in PBS for 1h. After blocking, the sections were incubated overnight at 4 °C with the PRAMEL1 and a mouse TUBB (T4026, Sigma-Adrich, St. Louis, MO) antibodies diluted (1:50 dilution each) in PBST (PBS containing 0.15% BSA, 0.1% Tween). Following the washing step (3× 5 min in PBS), sections were further incubated in the dark with FITC-conjugated donkey anti-goat-IgG (sc-2024, Santa Cruz Biotechnology, Santa Cruz, CA) and TRITC-conjugated donkey anti-mouse-IgG (sc-2300, Santa Cruz Biotechnology, Santa Cruz, CA) secondary antibodies diluted (1:100 dilution) in PBST for 1 h at RT. After secondary antibody incubation, sections were washed in PBS, and nuclei were counter stained with DAPI (Invitrogen, Carlsbad, CA) for 5 min at RT. Sections were then washed (3× 5 min) in PBS and coverslips mounted using the ProLong Gold antifade reagent (P36930, Invitrogen, Carlsbad, CA). Testis cross-sections incubated with peptide-preabsorbed PRAMEL1 antibody or secondary antibodies only were used as a negative control.

For immunofluorescence staining of squashed seminiferous tubules and epididymal sperm samples, slides were fixed with 4% paraformaldehyde for 20–30 min at RT and then permeabilized with ice-cold methanol (−20 °C) for 5 min. After washing in PBS, antigen retrieval was achieved by boiling in sodium citrate buffer for 20 min. Samples were then blocked for non-specific binding by incubating in PBS with 10% normal donkey serum for 1 h at RT. Following the washing steps, samples were stained for PRAMEL1 and TUBA using the PRAMEL1 and mouse TUBA (T6199, Sigma-Adrich, St. Louis, MO) (both diluted 1:50 in PBT) primary antibodies, respectively, and FITC-conjugated donkey anti-goat IgG and TRITC-conjugated donkey anti-mouse IgG (both diluted 1:100 in PBST) secondary antibodies. Cell nuclei were counterstained with DAPI and the coverslips were mounted as described above for testis cross-sections. All the samples were observed at RT using an Olympus BX51 (Olympus Optical Co. Ltd, Tokyo, Japan) epifluorescence microscope and digital images were captured with an Olympus DP71 microscope camera (Olympus America Inc., Center Valley, PA). Adobe Photoshop software (Adobe Inc., San Jose, CA) was used to assemble images into figures. No post-acquisition modifications were made to the original images.
